# Coagulation and Blood Factors and Clinical Disease Indicators in Patients with Chronic Angioedema and Urticaria—A Validation Study

**DOI:** 10.3390/biomedicines14040937

**Published:** 2026-04-20

**Authors:** Maja Štrajtenberger, Marinko Artuković, Iva Bešlić, Morana Belovic, Ivica Lokner, Liborija Lugović-Mihić

**Affiliations:** 1 Department of Pulmonology, Special Hospital for Pulmonary Diseases, 10000 Zagreb, Croatia; ivica.lokner@pulmologija.hr; 2 Nursing Department, University North, 42000 Varaždin, Croatia; marinko.artukovic@gmail.com; 3 Polyclinic Vita, 10000 Zagreb, Croatia; 4 Dom Zdravlja Zagreb—Zapad, 10000 Zagreb, Croatia; moranabelovic@gmail.com; 5 Department of Dermatology, University Hospital Center Sestre Milosrdnice, 10000 Zagreb, Croatia; liborija@gmail.com; 6 School of Dental Medicine, University of Zagreb, 10000 Zagreb, Croatia

**Keywords:** coagulation factors, serum blood cell factors, clinical disease indicators, angioedema, urticaria

## Abstract

**Background:** The interaction between coagulation pathways, inflammatory markers, and hematological parameters has not been sufficiently clarified in patients with chronic angioedema (AE) and urticaria. This study aimed to validate previously observed associations and to further explore their relationship with clinical disease control. **Methods:** In this cross-sectional validation study, 102 participants were enrolled and stratified into three groups: isolated AE (n = 33), AE associated with urticaria (AE/Urt; n = 34), and healthy controls (n = 35). Serum levels of coagulation factors (D-dimer, fibrinogen, factor VII), inflammatory markers (C-reactive protein [CRP], erythrocyte sedimentation rate [ESR]), and complete blood count parameters were analyzed. Disease control was assessed using the Angioedema Control Test (AECT). Appropriate non-parametric statistical tests were applied. **Results:** Only D-dimer values differed significantly between groups and were higher in patients with AE/Urt than in controls. At the same time, D-dimers were significantly more often elevated in both AE groups than in healthy individuals. Additionally, CRP values in both AE groups were significantly more often elevated than in controls, with significantly higher values in both AE groups (in both groups 85%) than in controls (57%). Coagulation markers and CRP demonstrated a positive correlation with age (r = 0.268–0.392; *p* ≤ 0.007), with fibrinogen of all coagulation markers showing the strongest age dependency (r = 0.334; *p* = 0.001). Gender-related differences in coagulation parameters were not statistically significant, although elevated fibrinogen levels were more common in male participants (*p* = 0.030). Disease control did not correlate linearly with any inflammatory markers, coagulation factor, age or gender. **Conclusions**: The findings support a contributory role of coagulation pathway activation and systemic inflammation in the pathophysiology of chronic angioedema and urticaria. These mechanisms may influence clinical disease expression and could represent potential targets for improved diagnostic stratification and therapeutic approaches. However, the interpretation of the present results should be approached with caution in light of several important study limitations, including demographic heterogeneity between the study groups and the relatively limited sample size.

## 1. Introduction

The understanding of the pathophysiology of angioedema (AE) has significantly advanced over the past decade, particularly in hereditary angioedema (HAE), in which, in addition to mutations in the gene encoding C1-esterase inhibitor, several novel mutations have been identified in proteins involved in blood enzyme cascades (e.g., factor XII, plasminogen, and angiopoietin). The deficiency or dysfunction of specific regulatory enzymes disrupts the balance between interconnected plasma cascade systems, including the kallikrein–kinin system, the renin–angiotensin system, the contact activation pathway of coagulation, fibrinolysis, and the complement pathway. This imbalance results in excessive bradykinin production, leading to increased vascular permeability and the clinical manifestation of angioedema.

In contrast, histamine-mediated angioedema is primarily driven by immune mechanisms, including allergic or autoimmune reactions that induce mast cell and basophil activation with subsequent release of histamine and other vasoactive mediators. These processes lead to vasodilation and increased vascular permeability through pathways distinct from bradykinin-mediated forms. Nevertheless, increasing evidence suggests that inflammatory and coagulation pathways interact closely in both histaminergic angioedema and chronic urticaria (CU).

The involvement of coagulation mechanisms in the pathogenesis of chronic urticaria, with or without associated angioedema, has been recognized for many years, and therapeutic attempts aimed at modulating these pathways have been reported. Clinical observations indicate beneficial effects of coagulation blockade in hereditary angioedema, particularly with antifibrinolytic agents such as tranexamic acid. However, the mechanisms by which coagulation factors contribute to the activation of cutaneous mast cells and circulating basophils in chronic non-hereditary angioedema remain insufficiently clarified. Similarly, the biological mechanisms underlying the occurrence of isolated angioedema compared with angioedema associated with urticaria (AE/Urt) are not fully understood.

Although the overall pathogenesis of chronic urticaria and chronic angioedema is complex and incompletely elucidated, current evidence highlights several important contributors. These include IgE- and IgG-mediated autoantibody responses leading to mast cell and basophil activation, activation of the coagulation and complement cascades, and interactions with infectious agents, cytokines, and other immunological mediators [1,2,3]. In clinical practice, reliable biomarkers that could differentiate hereditary from non-hereditary forms of angioedema are still lacking [4], as are markers capable of distinguishing between patients with chronic AE/Urt) and patients with isolated AE.

Both clinical entities have been associated with elevated systemic inflammatory markers, including C-reactive protein (CRP) and erythrocyte sedimentation rate (ESR), particularly during periods of active disease, while significantly lower levels are typically observed during remission [5,6]. In angioedema associated with urticaria, activation of skin mast cells via the extrinsic coagulation pathway has been proposed as a key pathophysiological mechanism. This activation may be driven by a combined effect of infectious stimuli, histamine release, and tissue factor-inducing cytokines such as tumor necrosis factor alpha (TNF-α), interleukin-1 beta (IL-1β), vascular endothelial growth factor (VEGF), and interleukin-33 (IL-33), which collectively contribute to endothelial dysfunction and increased vascular permeability [1,7,8,9,10,11,12].

Several studies have demonstrated elevated circulating levels of coagulation-related biomarkers in patients with chronic spontaneous urticaria (CSU), including D-dimers, fibrin, prothrombin fragments 1 + 2, CRP, and various pro-inflammatory cytokines such as TNF-α, transforming growth factor beta (TGF-β), IL-1, IL-6, IL-17, IL-31, and IL-33 [11,12,13]. Previous research has also suggested a positive correlation between disease severity and plasma D-dimer concentrations in patients with AE/Urt [9]. These findings support the hypothesis that activation of the coagulation pathway and fibrinolytic system contributes to thrombin generation and subsequent vascular changes that may exacerbate inflammatory responses.

The interplay between inflammation and coagulation represents a self-perpetuating biological loop capable of amplifying disease activity [14]. Coagulation activation may exert both local pathogenic effects, such as the development of inflammatory skin lesions, and systemic effects, including an increased risk of thrombotic events. For example, eosinophils within the cutaneous microenvironment of patients with CSU and angioedema may express tissue factor, thereby directly promoting coagulation and enhancing vascular permeability through mediator release. Enhanced thrombin generation further increases endothelial permeability and sustains the inflammatory cascade characteristic of CU and AE. Additionally, several reports have described cardiovascular complications, including myocardial infarction, during acute urticaria episodes, highlighting the potential systemic consequences of coagulation activation [15,16,17,18].

Despite substantial advances in understanding the pathophysiology of AE, many aspects of the underlying mechanisms remain unresolved. In particular, the differential role of coagulation activation in isolated AE compared with AE/Urt requires further investigation. Clarifying these mechanisms may contribute to improved diagnostic stratification and the identification of novel therapeutic targets.

Therefore, the aim of the present study was to investigate the relationship between coagulation factors and other serum biomarkers in relation to the occurrence and clinical presentation of AE and CU. Special emphasis was placed on identifying potential alterations in haemostatic parameters that may reflect involvement of the coagulation system in disease pathophysiology. By analyzing differences between affected patients and healthy controls, as well as among distinct clinical subtypes, the study sought to determine whether specific laboratory markers could serve as potential diagnostic or prognostic indicators and thereby enhance understanding of the biological mechanisms underlying symptom development.

## 2. Materials and Methods

### 2.1. Patient Population

This cross-sectional validation study included a total of 102 participants divided into three groups: 33 patients with isolated chronic angioedema (AE), 34 patients with chronic angioedema associated with urticaria (AE/Urt), and 35 healthy controls [19]. All patients were examined at the Special Hospital for Pulmonary Diseases in Zagreb by a specialist in allergology and clinical immunology.

Following a detailed clinical evaluation, all participants were informed about the study protocol, which was voluntary and anonymous, and written informed consent was obtained. Peripheral venous blood samples were collected to determine serum inflammatory parameters (complete blood count, erythrocyte sedimentation rate [ESR], and C-reactive protein [CRP]) and coagulation factors (D-dimer, fibrinogen, and factor VII). Disease control was assessed using the validated Angioedema Control Test (AECT) questionnaire. Participants were given the opportunity to request additional clarification while completing the questionnaire.

Diagnostic evaluation for chronic angioedema subtypes:

All patients underwent a standardized diagnostic workup to exclude non-histaminergic forms of angioedema. To rule out hereditary angioedema (HAE), complement testing was performed in all cases, including serum C4 levels and both quantitative and functional C1-inhibitor (C1-INH) assays. Patients with decreased C4, reduced C1-INH concentration, or impaired C1-INH function were excluded. Angiotensin-converting enzyme inhibitor (ACEi)-induced angioedema was excluded through systematic review of medication history; patients receiving ACEi therapy at the time of symptom onset, or within the relevant pharmacokinetic timeframe, were not eligible. In addition, individuals with recurrent angioedema episodes that persisted despite ≥3 months of ACEi discontinuation were excluded if ACEi involvement could not be confidently ruled out. Only patients with normal complement studies, no ACEi exposure, and clinical features consistent with histaminergic angioedema were included in the AE and AE/Urt groups.

Inclusion criteria were adult patients diagnosed with chronic angioedema according to current international guidelines who provided informed consent [19]. Exclusion criteria included isolated chronic urticaria without angioedema, use of specific medications within the preceding 15 days (antimicrobial therapy, antineoplastic treatment, systemic corticosteroids, or immunosuppressive therapy), active smoking, known autoimmune or malignant disease, diabetes mellitus, upper respiratory tract infection within the previous 30 days, ongoing anticoagulant therapy, pregnancy, and breastfeeding. As noted above, patients with laboratory or clinical findings consistent with HAE or ACEi-related angioedema were also excluded.

### 2.2. Assessment of AE Severity—Angioedema Control Test (AECT)

The Angioedema Control Test (AECT) is a validated patient-reported outcome questionnaire designed to assess disease control in patients with recurrent angioedema. Current clinical guidelines recommend its use for evaluating disease activity, the impact on quality of life, and overall disease control as part of standardized patient-reported outcome measures (PROMs) [19,20]. The AECT was developed in response to the lack of adequate tools for assessing angioedema control [21,22,23]. Validation studies have demonstrated that the AECT is a reliable and clinically useful instrument for monitoring disease control and facilitating therapeutic decision-making. A total score of less than 10 indicates insufficient disease control, whereas a score of 10 or higher reflects well-controlled disease [24].

The questionnaire is simple to administer and comparable to other disease control instruments, such as the Asthma Control Test and the Urticaria Control Test. It can be applied in both routine clinical practice and clinical research settings; however, it should be complemented by additional measures assessing disease activity and quality of life [25,26,27].

### 2.3. Assessment of Serum/Blood Cells

C-reactive protein (CRP) levels were measured using a Cobas Integra 400 Plus biochemical analyser (Roche Diagnostics, Vienna, Austria) in accordance with the manufacturer’s protocols and reagents. Serum CRP concentrations were determined by latex particle immunoturbidimetry, based on the principle of CRP binding to latex particles coated with monoclonal anti-CRP antibodies. Changes in turbidity of the reaction mixture were measured at a wavelength of 552 nm. The method is standardized according to the IFCC reference preparation CRM470 (RPPHS 91/0619) for plasma proteins.

Complete blood count parameters were analyzed using an Advia 2120 hematology analyser (Siemens, Munich, Germany) with original manufacturer reagents. Cellular particle concentration was determined by flow cytometry combined with spectrophotometric measurement using potassium cyanide methodology.

Hemoglobin concentration was measured colorimetrically at a wavelength of 546 nm. The analytical process involves erythrocyte lysis by surfactant and subsequent denaturation of hemoglobin. Iron within the haem molecule is oxidized from the ferrous to the ferric state through the action of cyanide, resulting in a measurable change in absorbance.

The erythrocyte sedimentation rate (ESR) was determined using the standard Westergren method. Standardized graduated pipettes (BD, Becton Dickinson, Drogheda, Ireland) and citrated whole-blood samples were used, and the height of the plasma column was recorded after one hour of vertical sedimentation.

### 2.4. Assessment of Serum Coagulation Factors

Venous blood samples were collected into evacuated tubes containing trisodium citrate (BD, Becton Dickinson, Drogheda, Ireland) anticoagulant at a ratio of nine parts blood to one part 3.8% (*w*/*v*) citrate solution. After centrifugation (Andreas Hettich GmbH, Tuttlingen, Baden-Wurttemberg, Germany) for 15 min at 1500× *g*, platelet-poor plasma was separated, stored at −80 °C, and thawed at 37 °C immediately before laboratory analysis of coagulation parameters (factor VII activity, fibrinogen concentration, and D-dimer levels).

D-dimer concentrations were measured using the Cobas Integra 400 Plus analyser (Roche Diagnostics, Vienna, Austria) with original manufacturer reagents and protocols. Quantification was performed by latex immunoturbidimetric assay using the D-dimer reagent cassette D-DI2 and the D-dimer Gen.2 calibrator set. The method is standardized against the Asserachrom D-dimer reference procedure.

Factor VII activity and fibrinogen concentration were determined using a BCS XP coagulation analyser (Siemens Healthineers, Marburg, Germany) with manufacturer-provided reagents. Fibrinogen levels were measured by the Clauss clotting method using the Multifibren U reagent (Simens Healthcare Diagnostic Products GmbH, Marburg, Hessen, Germany). Factor VII activity was assessed by a coagulometric method employing factor VII-deficient plasma. Reaction mixtures consisting of patient plasma and factor VII-deficient plasma (Simens Healthcare Diagnostic Products GmbH, Marburg, Hessen, Germany) were tested using Thromborel S reagent (Simens Healthcare Diagnostic Products GmbH, Marburg, Hessen, Germany), and results were expressed as percentages based on a calibration curve generated with serial dilutions of Standard Human Plasma (Simens Healthcare Diagnostic Products GmbH, Marburg, Hessen, Germany). The calibration material is traceable to the International Standard WHO 09/172.

### 2.5. Statistical Data Processing

Normality of data distribution was evaluated using the Kolmogorov–Smirnov test. As most variables did not demonstrate normal distribution, non-parametric statistical tests were applied. The Kruskal–Wallis test was used to compare age distribution and coagulation factor values among study groups, followed by post hoc pairwise comparisons using the Mann–Whitney U test with Bonferroni correction.

Effect sizes were calculated as r = z/√N for the Mann–Whitney test and ε^2^ = H/[(n^2^ − 1)/(n + 1)] for the Kruskal–Wallis test. Interpretation followed Cohen’s criteria, whereby r values of 0.25–0.30 indicated a small effect size, 0.30–0.50 a moderate effect, 0.50–0.70 a large effect, and values greater than 0.70 a very large effect. For ε^2^ values, squared Cohen thresholds were applied (0.06–0.09 small, 0.09–0.25 moderate, 0.25–0.49 large, and >0.49 very large).

The χ^2^ test was used to compare categorical variable frequencies, with effect size quantified using Cramer’s V and interpreted according to Cohen-based criteria. Spearman’s rank correlation analysis was performed to evaluate associations between continuous variables.

All statistical analyses were conducted using commercial statistical software (IBM SPSS Statistics for Windows, version 22.0; IBM Corp., Armonk, NY, USA).

## 3. Results

### 3.1. Analysis of the Tested Sample

The sample included 102 participants aged between 19 and 81 years (median 49, interquartile range 36–58), 81% of which were female. The participants were divided into three groups: 33 patients with isolated AE (AE), 34 patients with AE/Urt (CU + AE), and 35 healthy individuals (controls). The description of the sample is presented in Table 1.

**Figure 1:**
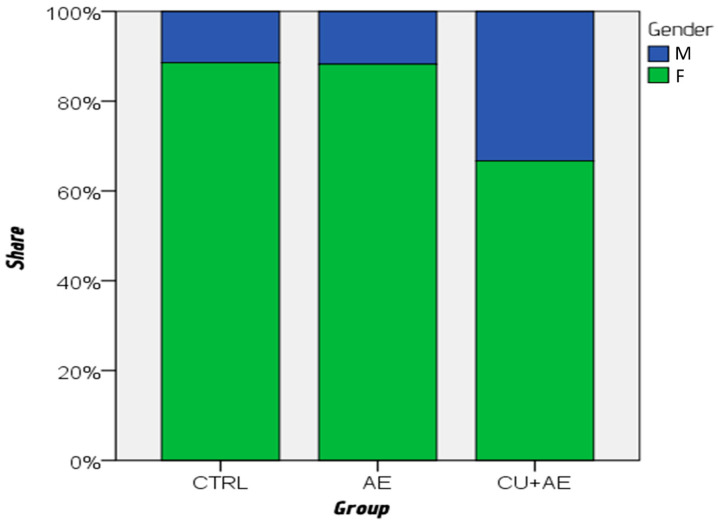
Gender distribution by group, compared by χ^2^ test.

**Figure 2:**
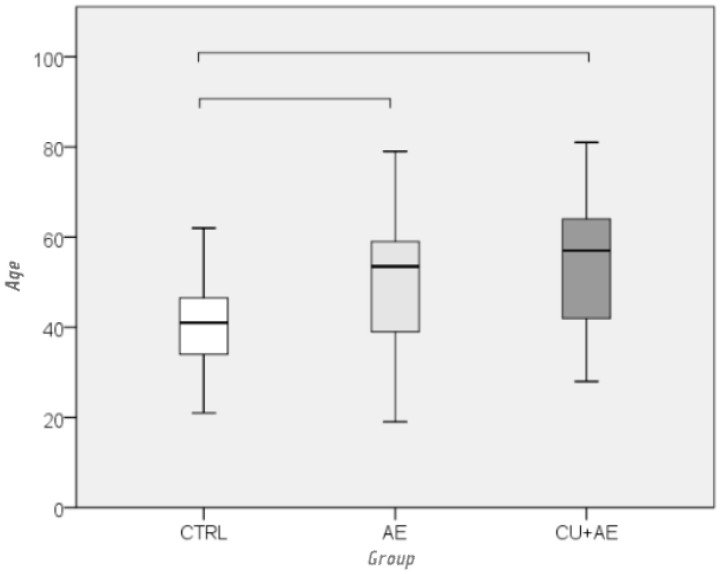
Comparison of age between groups by Kruskal–Wallis and Mann–Whitney post hoc test. Groups connected by horizontal lines are statistically significantly different.

Women were found to be significantly younger than men (median age: 45 vs. 60 years), with a moderate effect size (*p* = 0.002; r = −0.311). The gender distribution differed between the three groups (*p* = 0.022), with more men in the AE/Urt (CU + AE) group (11/22; 33%) than in those with AE alone (AE) (4/34; 12%) and healthy controls (CTRL) (4/35; 11%) (Figure 1). The groups also differed by age, with a moderate effect size (*p* < 0.001; ε^2^ = 0.153) (Figure 2); the control group was much younger than patients with isolated AE (AE) (*p* = 0.026) and patients in the AE/Urt group (CU + AE) (*p* < 0.001).

### 3.2. Results of Coagulation Factor Analysis

**Figure 3:**
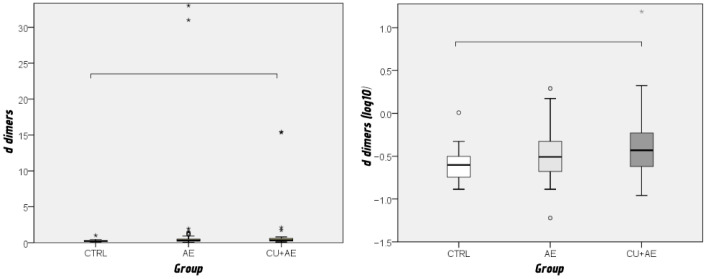
Comparison of D-dimer between groups (real values shown on the left and log-transformed values on the right) by Kruskal–Wallis and Mann–Whitney post hoc test. Groups connected by a horizontal line are statistically significantly different. Cicrcles indicate mild outliers. Asteriks represents extreme outliers.

Among the coagulation factors, only D-dimer levels showed a significant difference between groups (*p* = 0.009; ε^2^ = 0.092), with higher values observed in patients with AE/Urt (CU + AE) compared to the control group (CTRL) (*p* = 0.012) (Figure 3).

**Figure 4:**
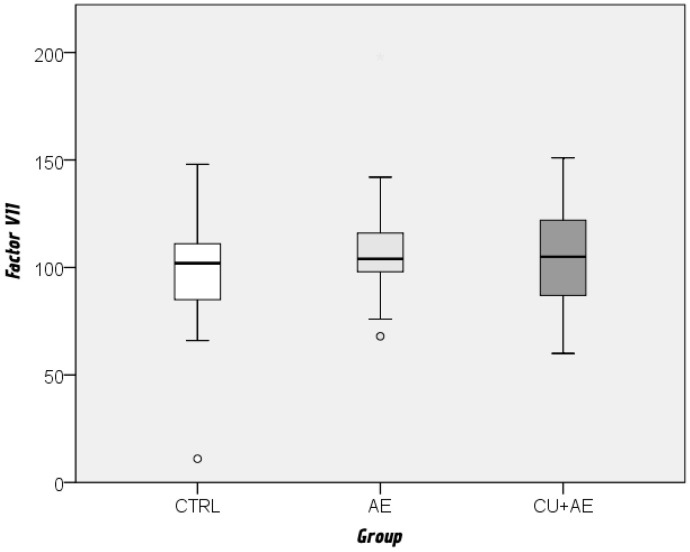
Comparison of factor VII levels between groups by Kruskal–Wallis and Mann–Whitney post hoc test. Cicrcles indicate mild outliers.

**Figure 5:**
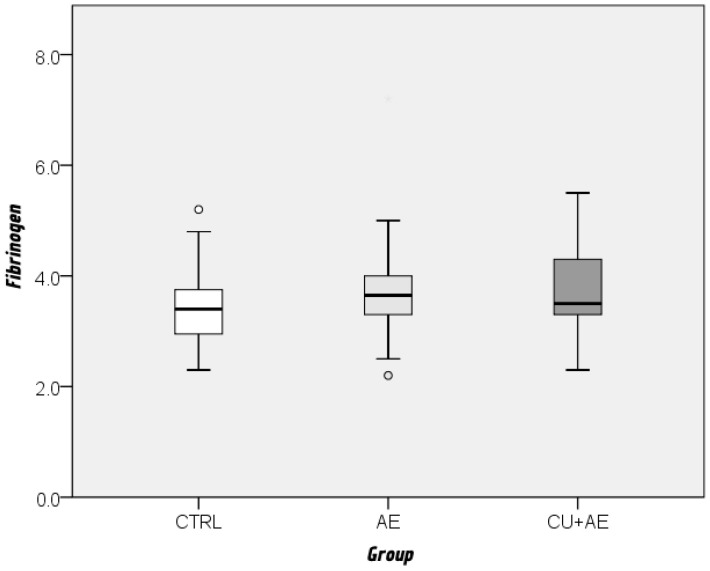
Comparison of fibrinogen levels between groups by Kruskal–Wallis and Mann–Whitney post hoc test. Cicrcles indicate mild outliers.

Fibrinogen and factor VII values did not differ significantly between the three groups (Figure 4 and Figure 5).

### 3.3. Results of Analysis of Other Tested Serum Factors

CRP values were significantly higher in patients with isolated AE (0.007) as well as AE/Urt (*p* = 0.006) than in controls.

**Figure 6:**
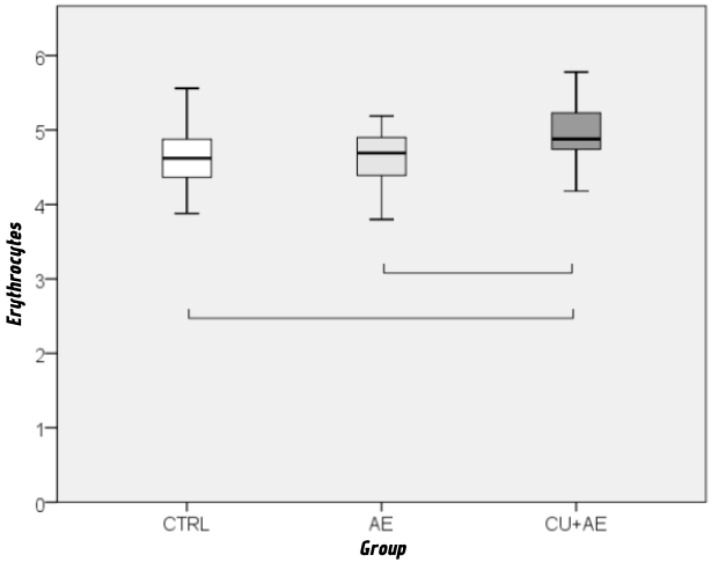
Comparison of erythrocyte levels between groups by Kruskal–Wallis and Mann–Whitney post hoc test. Groups connected by a horizontal line are statistically significantly different.

**Figure 7:**
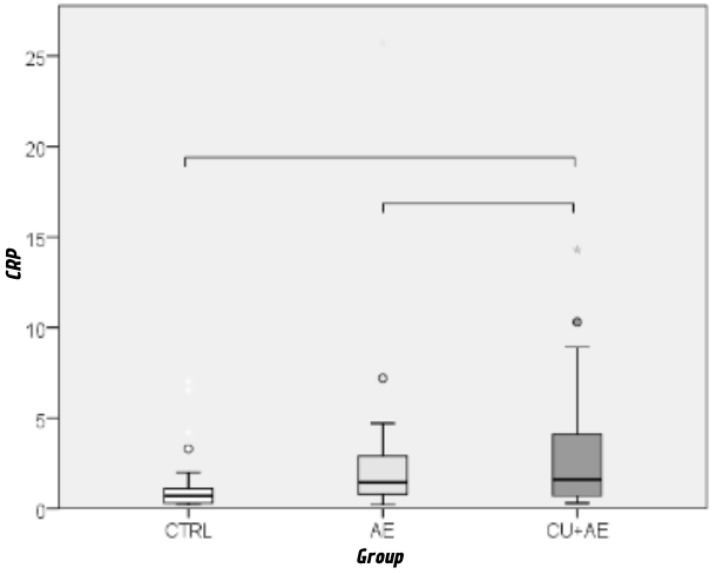
Comparison of CRP levels between groups by Kruskal–Wallis and Mann–Whitney post hoc test. Circles indicate outliers, and asterisks indicate extremes. Groups connected by a horizontal line are statistically significantly different. Cicrcles indicate mild outliers. Asteriks represents extreme outliers.

Furthermore, analysis of these factors has shown a significant difference in the levels of erythrocytes (*p* = 0.003; ε^2^ = 0.117) and CRP (*p* = 0.002; ε^2^ = 0.125) (Figure 6 and Figure 7) between AE/Urt group and other two patient groups, with a moderate effect size. There were no differences in the levels of other analyzed biomarkers.

### 3.4. Results of Disease Control Factors Testing

**Figure 8:**
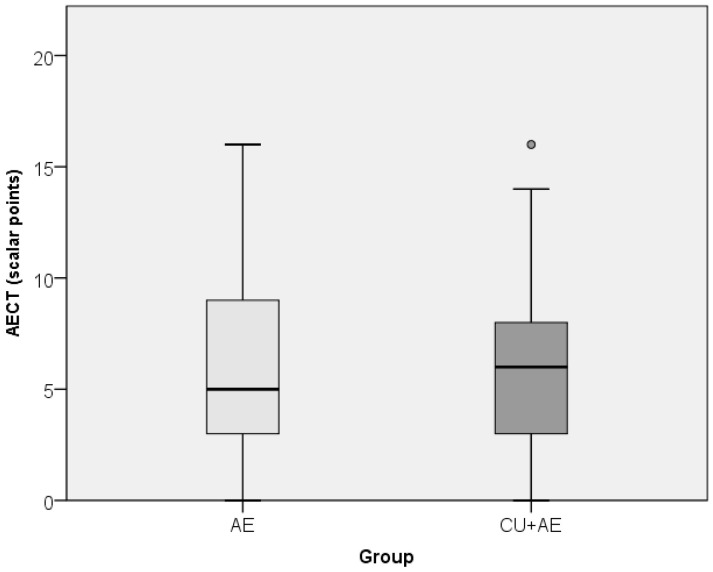
Comparison of disease control intensity between groups by Mann–Whitney test. Cicrcles indicate mild outliers.

**Figure 9:**
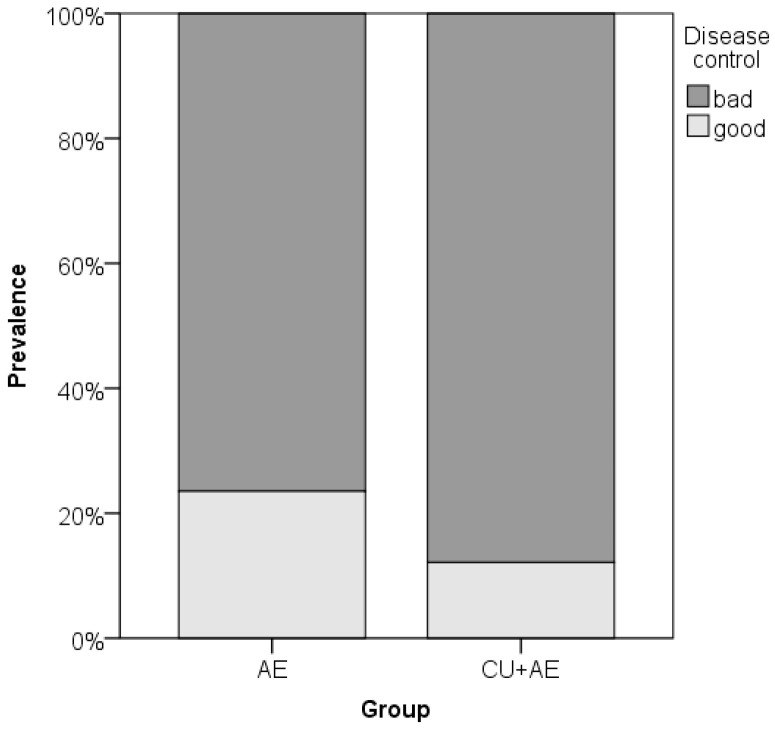
Comparison of disease control prevalence between groups by Fischer exact test.

Data on disease control (its intensity and prevalence) did not differ between the groups of patients (Figure 8 and Figure 9). Disease control did not correlate with any inflammatory markers, coagulation factor, age or gender (Table 2).

The correlation of individual factors with the age of the participants was determined. Thus, coagulation factors correlated with age (linear, positive and weak to moderate correlation). Values of factor VII (r = 0.268; *p* = 0.007), D-dimer (r = 0.309; *p* = 0.002) and fibrinogen (r = 0.334; *p* = 0.001) all increased with age, with fibrinogen showing the strongest dependence on age. CRP values also correlated positively with age (r = 0.392 *p* < 0.001), while platelet levels (r = −0.313; *p* = 0.001) correlated negatively. The correlation was weak to moderate.

When groups are compared using a 50-year cutoff, it was found that the following biomarker levels differed significantly: D-dimer, CRP, ESR, salivary HBD2, fibrinogen, factor VII, erythrocytes, and platelets (*p* ≤ 0.035; r = −0.209 to −0.438). Platelet counts were lower in the older group, whereas all other biomarkers were higher. In participants aged ≤ 49 years, no differences in biomarkers or disease activity were observed among the three groups. In participants aged ≥ 50 years, a difference among the three groups was found only for erythrocyte count (*p* = 0.013), with the AE group showing significantly lower values compared to the CU + AE group (*p* = 0.013; r = −0.450).

By gender, there were no significant differences in the levels of coagulation factors between genders, but elevated fibrinogen values were observed more frequently in men (5/19; 26%) than women (6/83; 7%), with a small effect size (*p* = 0.030; V = 0.240). Men also had higher erythrocyte (*p* < 0.001) and CRP levels (*p* = 0.005). Similarly, men had lower platelet (*p* = 0.001) and ESR levels (*p* = 0.004).

## 4. Discussion

In our recent pilot study, the first to compare coagulation factor values among patients with isolated angioedema (AE), angioedema with urticaria (AE/Urt), and healthy controls [13], increased coagulation propensity was demonstrated only in patients with isolated AE. These findings confirmed earlier reports [9,28]. In the present validation study, however, only D-dimer levels were significantly higher in patients with AE/Urt compared with controls. Nevertheless, D-dimer values were more frequently elevated in both AE subgroups than in controls. The observed difference disappeared after stratification by age. Although D-dimer levels have been widely studied in chronic spontaneous urticaria (CSU), only a few investigations have evaluated them in angioedema. Three of these focused on hereditary angioedema (HAE). Apart from our pilot study, no previous research has specifically analyzed D-dimer levels in isolated AE.

Comparison of the pilot and validation phases reveals important differences. These reflect the larger sample size and the broader analytical design of the validation study. While the pilot phase showed elevated D-dimer and fibrinogen levels, particularly in patients with concomitant urticaria, the validation study confirmed D-dimer as the only consistently increased coagulation marker. The expanded analytical panel, including CRP, ESR, complete blood count parameters, and AECT assessment, provided a more comprehensive overview of disease activity. CRP values differed significantly from controls, whereas other coagulation markers did not. This finding also disappeared when stratification by age was done. The lack of fibrinogen significance in the validation phase may be explained by increased heterogeneity and reduced influence of outliers. Age-related effects also became clearer. Fibrinogen showed strong age dependency and increased across all groups. Sex-related differences were modest. Elevated fibrinogen values were more frequent in men, but overall coagulation markers did not differ significantly by sex. Thus, the validation study strengthens the pilot findings by confirming D-dimer elevation and provides a more stable interpretation of coagulation and inflammatory changes in angioedema.

Previous CSU studies consistently demonstrated elevated D-dimer levels compared with controls [5,12,29,30,31,32,33,34,35,36,37]. Many also reported correlations between D-dimer levels and disease severity, as well as improvement after effective therapy [5,8,29,38,39,40,41,42]. Our findings are consistent with these observations. Increased plasma D-dimer levels in patients with AE support activation of coagulation and fibrinolysis. This activation may contribute to thrombus formation and increased cardiovascular risk.

Several earlier investigations confirmed coagulation system activation in CSU and AE. These conclusions were based on analyses of fibrinogen, factor VII, platelet count, and prothrombin fragment (PF1 + 2). One study demonstrated significantly increased values of multiple coagulation markers, including fibrinogen, in CSU patients [43]. Our results did not confirm these findings. Nevertheless, previous authors suggested that anticoagulant therapy could be considered in selected AE patients and emphasized the need for further research in this area [43].

Age showed a positive linear association with coagulation factor levels in our cohort. This relationship ranged from weak to moderate strength. Fibrinogen was the most age-dependent parameter. Factor VII and D-dimer levels also increased with age. These findings align with previous studies demonstrating age-related increases in multiple coagulation factors, von Willebrand factor, thrombin generation, and platelet activation [44,45,46,47].

Older patients with CSU have been reported to have a higher incidence of cardiovascular disease. This may partly result from hypercoagulability associated with elevated fibrinogen and coagulation factor levels. Such imbalance in haemostasis is an important contributor to cardiovascular risk [45]. Similarly, venous thromboembolism incidence increases with age [44]. Additional contributors include comorbidities, endothelial dysfunction, platelet alterations, and changes in anticoagulant pathways. Together, these factors support the concept of ageing as an acquired prothrombotic state [47].

CRP values were significantly more often elevated in both AE subgroups than in controls. Approximately 85% of patients with isolated AE and AE/Urt had elevated CRP levels, compared with 57% of controls. A positive linear correlation between CRP and age was also observed. These findings are consistent with previous reports showing increased CRP levels in CSU and AE populations [5,42,48,49,50]. Several studies also demonstrated correlations between CRP and disease severity or activity scores [28,51,52].

Elevated ESR values were observed in only a small proportion of our patients. These increases were slightly more frequent in isolated AE. However, overall ESR findings differed from many earlier studies reporting significantly increased ESR in CSU and AE. Literature data remain inconsistent. Some prospective studies have shown similar ESR and CRP values in CSU and idiopathic recurrent histaminergic angioedema [53], which is consistent with our observations.

Erythrocyte counts were elevated in a minority of subjects. Higher values were more common in the AE/Urt group, although overall differences were not statistically significant. Nevertheless, AE/Urt patients showed significantly higher erythrocyte levels than controls and isolated AE patients. Only a limited number of studies have evaluated erythrocyte counts in CSU. Most did not demonstrate associations with disease severity or response to omalizumab therapy [54].

In our cohort, CRP correlated positively with age, whereas platelet counts showed a weak negative association. Men more frequently had elevated fibrinogen levels, higher erythrocyte counts, and higher CRP values. Conversely, platelet counts and ESR were lower in men. Previous studies indicate that CRP increases with age in healthy populations [55,56]. Women usually have higher CRP levels than men [57], which was not observed in our study. This discrepancy suggests disease-specific influences on inflammatory markers in AE. Age-adjusted analyses also showed interactions between sex and AE/Urt status for factor VII levels.

Our study revealed a significant association between age and factor VII levels, which supported previous studies [46,47]. In our study, fibrinogen values were mostly dependent on age. Therefore, our results are consistent with previous studies.

Basophil counts were reduced in most patients. Basopenia was observed in 93% of AE/Urt patients and 88% of isolated AE patients. Previous prospective studies identified basophil levels, sex distribution, and IgE receptor antibody status as key pathogenetic differences between chronic histaminergic angioedema and CSU, which we did not determine in our study [53]. Basophil counts are known to correlate negatively with CSU activity. Therefore, their assessment has been recommended as part of diagnostic evaluation [19,58,59,60]. Basopenia likely reflects recruitment of basophils to inflamed skin sites and altered IgE-mediated signalling pathways.

Leukocyte levels were more frequently elevated in AE/Urt patients. However, previous CSU studies did not demonstrate strong associations between leukocyte count and disease activity. Neutrophil elevations were mild and slightly more frequent in AE/Urt patients, supporting earlier findings [48]. Platelet abnormalities were uncommon. Mild thrombocytopenia occurred somewhat more often in isolated AE. Platelet counts showed weak negative correlations with age and were lower in male patients. Although platelets are involved in inflammatory and immune processes, their exact pathogenetic role in CSU and AE remains unclear. Further studies are required.

Angioedema control did not differ significantly between patient subgroups. Nevertheless, poorer control was somewhat more frequent in AE/Urt patients. No linear correlations were observed between disease control and biomarkers, age, or sex. Multivariable regression analysis identified sex and erythrocyte count as independent predictors of disease control. Better control was observed in men and in patients with lower erythrocyte levels. This model explained approximately 23% of the variance.

This study has several limitations. The relatively modest sample size may have reduced statistical power. Age and sex distribution were not fully balanced. The single-centre design may also limit generalizability. Larger multicentre longitudinal studies are needed to confirm these findings. Finally, the cross-sectional design precludes assessment of causality.

In hereditary angioedema, antifibrinolytic therapy such as tranexamic acid has demonstrated clinical benefit. However, the mechanisms linking coagulation activation with mast cell and basophil activation in chronic non-hereditary AE remain unclear. The potential role of anticoagulant therapy therefore warrants further investigation, particularly in patients with additional cardiovascular risk factors. Previous reports describe disease remission following treatment with heparin, warfarin, or combination anticoagulant approaches in selected CSU cases [61,62,63,64,65,66,67,68,69]. These observations highlight the importance of coagulation cascade activation in chronic AE and CSU and support future therapeutic research.

## 5. Conclusions

In conclusion, the present study demonstrated statistically significant differences in D-dimer levels among the analyzed groups, further supporting the hypothesis that activation of the coagulation cascade may play a relevant role in the underlying pathophysiological mechanisms of angioedema and urticaria. These findings are in line with the growing body of evidence suggesting a complex interplay between inflammatory and hemostatic pathways, which may contribute to endothelial dysfunction and the vascular alterations characteristic of these conditions. However, the interpretation of the present results should be approached with caution in light of several important study limitations, including the demographic heterogeneity between the study groups and the relatively limited sample size. These factors may have influenced the statistical power of the analyses and potentially restrict the external validity and broader generalizability of the observed associations.

## Figures and Tables

**Table 1:** Sample description.

Variable	Average	Std. Deviation	Minimum	Maximum	Median	IQR
Age	47.7	14.3	19	81	49	36–58
Factor VII	104.4	24.3	11	198	104	88.8–118.3
Fibrinogen	3.6	0.8	2.2	7.2	3.5	3.2–4.0
D-dimers	1.31	4.85	0.06	33.00	0.29	0.3–0.44
Erythrocytes	4.8	0.4	3.8	5.8	4.8	4.5–5.0
Platelets	255.5	57.5	145	492	258.5	220.0–2 84.3
Neutrophils	4.1	2.2	1.8	18.6	3.6	2.9–4.7
Basophils	0.04	0.05	0.00	0.39	0.03	0.02–0.04
Leucocytes	6.5	2.5	3.2	22.3	6.2	4.9–7.1
ESR	10.2	8.0	2	40	7.0	4.8–13.3
CRP	2.4	3.7	0.2	25.7	1.1	0.6–2.7
AECT score	6.4	4.5	0	16	5	3–8

IQR—interquartile range.

**Table 2:** Correlation between clinical disease control (AECT) and serum levels of coagulation factors, inflammatory markers, and complete blood count parameters, assessed by Spearman correlation.

		AECT Score
Factor VII	r	0.135
	*p*	0.278
Fibrinogen	r	−0.003
	*p*	0.983
Leucocyts	r	−0.207
	*p*	0.094
Erytrocytes	r	−0.179
	*p*	0.147
Platlets	r	0.02
	*p*	0.874
Neutrophyls	r	−0.249
	*p*	0.043
Basophils	r	0.005
	*p*	0.969
ESR	r	0.11
	*p*	0.378
D dimer	r	0.042
	*p*	0.734
CRP	r	−0.06
	*p*	0.631

## Data Availability

The original contributions presented in this study are included in the article. Further inquiries can be directed to the corresponding author.
